# Curcumin inhibits the proteolytic process of SREBP‐2 by first inhibiting the expression of S1P rather than directly inhibiting SREBP‐2 expression

**DOI:** 10.1002/fsn3.1985

**Published:** 2020-11-08

**Authors:** Yongnan Li, Shuodong Wu

**Affiliations:** ^1^ The Sixth General Surgery Biliary & Vascular surgery Shengjing Hospital of China Medical University Shenyang City China

**Keywords:** cholesterol gallstones, curcumin, NPC1L1, S1P, SREBP‐2

## Abstract

Many studies have demonstrated that curcumin can downregulate mRNA levels of sterol regulatory element‐binding proteins (SREBP‐2); however, our study did not find similar results. This study was designed to demonstrate that curcumin inhibits the proteolytic process of SREBP‐2 by first inhibiting the expression of membrane‐bound transcription factor site‐1 protease (S1P) rather than directly inhibiting SREBP‐2 expression. After curcumin treatment, Caco‐2 cells were collected to observe the dose‐ and time‐dependent dynamics of precursor and mature SREBP‐2, transcription factor‐specific protein 1 (SP‐1), and SREBP cleavage‐activating protein (SCAP). After curcumin treatment, SREBP‐2 distribution was detected in the cells and S1P protein expression was examined. Curcumin could downregulate mRNA levels of SREBP2, SP‐1 and SCAP, but it did not simultaneously downregulate the expression of precursor SREBP‐2 (pSREBP‐2) and SCAP. Curcumin can inhibit the proteolytic process of SREBP‐2, reduce the production of mature SREBP‐2 (mSREBP‐2), and change the cellular distribution of SREBP‐2. The inhibitory effect of curcumin on SP‐1 protein expression is short‐acting. Curcumin could downregulate the mRNA and protein expression of S1P, but has no obvious inhibitory effect on the mRNA and protein expression of S2P (site‐2 protease). Curcumin can inhibit the SREBP‐2 proteolytic process to reduce mSREBP‐2 which functions as a transcription factor, affecting the regulation of cholesterol metabolism‐related genes. Curcumin does not directly inhibit the expression of mSREBP‐2 protein, and it has no such inhibitory effect for at least a short period of time, although curcumin does reduce the amount of mSREBP‐2 protein. S1P is a key protease in the hydrolysis of pSREBP‐2 into mSREBP‐2. Therefore, curcumin may decrease the amount of mSREBP‐2 by directly inhibiting the expression of S1P mRNA and protein.

## Graphical abstract

This study found for the first time that although curcumin could indeed downregulate the expression of SREBP‐2 mRNA, it did not down‐regulate the expression of SREBP‐2 protein. This discovery is different from all previous studies on the relationship between curcumin and SREBP‐2, which is a new discovery. In addition, this study also found that curcumin inhibited the process of SREBP‐2 proteolysis, possibly by inhibiting the expression of S1P, which is also a new discovery and has not been reported.

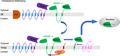

## 1 INTRODUCTION

Many studies have indicated that the formation of cholesterol gallstones is closely related to the absorption of cholesterol in the small intestine. A high‐fat diet can promote the formation of cholesterol stones and reducing cholesterol absorption in the small intestine can inhibit the formation of calculus. (Alrefai et al., 2007) The Niemann‐Pick C1‐like 1 (NPC1L1) protein of the intestinal epithelial brush‐border membrane is a cholesterol transporter (Altmann et al., 2004; Brown & Goldstein, 2007). Kumar et al (Brown et al., 2011) found that curcumin can inhibit NPC1L1 expression to reduce cholesterol absorption in Caco‐2 cells, which is mediated by SREBP‐2. We used a high‐fat diet mouse model to identify that curcumin can reduce the formation of cholesterol gallstones in a process regulated by SREBP‐2. (Ding et al., 2015).

SREBP‐2 is a member of the SREBP family which includes three proteins: SREBP‐1a, SREBP‐1c and SREBP‐2. SREBP‐2 is mainly involved in the activation of low‐density lipoprotein receptors and the expression regulation of cholesterol metabolism pathways related proteins. The SREBP‐2 precursor protein (precursor SREBP‐2, pSREBP‐2) is a membrane protein with a molecular weight of approximately 125 kDa which is localized on the endoplasmic reticulum as a hairpin structure and contains three domains: the N‐terminal (about 480 amino acids), C‐terminal (about 590 amino acids), and middle regions (about 80 amino acids) (Feng et al., 1999; Field et al., 2018). The C‐terminus of the pSREBP‐2 connects with the C‐terminus of the SREBP cleavage‐activating protein (SCAP) to form the SREBP/SCAP complex. The sterol‐sensing domain (SSD) consists of 2–6 transmembrane helices near the N‐terminus of SCAP. The SREBP/SCAP complex anchors to the endoplasmic reticulum by linking to the endoplasmic reticulum intrinsic structural protein insulin induced gene** **(INSIG) (Field et al., 2018). As sterol concentration decreases, the linkage between SCAP and INSIG disappears and the SCAP conformation changes, leading to the transport of the SREBP/SCAP complex from the endoplasmic reticulum to the Golgi apparatus (Field et al., 2018). In the Golgi body, site‐1 protease (S1P) cleaves SREBP‐2 protein in the hydrophilic ring of SREBP‐2. The site‐2 protease (S2P) cleaves the N‐terminal domains of SREBP‐2 across the Golgi membrane. The N‐terminal SREBP‐2 (mSREBP‐2, approximately 62 kDa) is then released into the nucleus to act as a transcription factor via its NH_2_‐terminal bHLH transcription factor domains (Feng et al., 1999; Field et al., 2018) (Sketch Map). Only mSREBP‐2 has transcription factor functions, not its precursor, and the two forms have different molecular weights.

Many studies have confirmed that curcumin can inhibit SREBP‐2 mRNA expression (Brown et al., 2017; Ding et al., 2016; Kang & Chen, 2009; Kang & Chen, 2009; Kumar et al., 2011; Li et al., 2015; Liu et al., 2017). This may depend on the inhibition of specificity protein‐1 (SP‐1) protein expression, which can bind to the GC‐box in the SREBP‐2 promoter sequence (Kang & Chen, 2009). Curcumin is reported to inhibit both the transcription and translation of SREBP‐2 (Kang & Chen, 2009; Li et al., 2016; Liu et al., 2017). Curcumin likely directly inhibits SREBP‐2 protein expression through inhibiting its transcription. Does curcumin downregulate the expression of SREBP‐2 mRNA and also reduce the expression of SREBP‐2 protein? Does curcumin interfere with the proteolysis process of SREBP‐2? If curcumin interferes with the proteolytic process of SREBP‐2, which step of this process may be affected? These questions are the main contents of this study.

## 2 MATERIALS AND METHODS

### 2.1 Cell culture

Human colorectal adenocarcinoma cell line (Caco‐2) was purchased from the Cell Bank of the Chinese Academy of Sciences, and cells were grown routinely in T‐75‐cm^2^ plastic flasks at 37°C in a 5% CO2%‐95% air environment. The culture medium consisted of MEM (Invitrogenz, 11090081), 20% FBS (Gibco), 1% Glutamax (Invitrogen, 35050061), 1% Nonessential Amino Acid (Invitrogen, 11140050), 1% Sodium Pyruvate 100 mM Solution (Invitrogen, 11360070), 100 IU/ml penicillin, and 100 μg/ml streptomycin (Life Technologies, Inc.). Cells from passages between 20 and 40 were plated in six‐well Falcon plates at a density of 2 × 10^4^ cells/cm^2^ and were fed with fresh incubation media every alternate day. Curcumin was obtained from Sigma (St. Louis, MO, Curcuminoid content ≥94% and Curcumin ≥80%). DMSO is used to dissolve curcumin (the concentration of DMSO was 0.1%). The selection of curcumin concentration was mainly based on reference 4.

### 2.2 RNA extraction and qPCR

Total RNA was prepared from Caco‐2 cells using an RNAiso Plus (TAKARA, 9108) according to the manufacturer's instructions. Equal amounts of RNA from both treated and control samples were reverse transcribed and amplified in one step reaction using 5 × PrimeScript RT Master Mix (TAKARA, RR036A). Real‐time PCR was performed using 2 × ChamQ Universal SYBR qPCR Master Mix (Vazyme Biotech Co., Ltd) with gene‐specific primers as follows: human SREBP‐2: Forward Primer CTCCATTGACTCTGAGCCAGGA; ReversePrimer GAATCCGTGAGCGGTCTACCAT human SCAP: Forward Primer TCACGTTGCAGCCGTCTTCCTT; Reverse Primer CAGGATGCCAATCCAGACAACG human SP‐1: Forward PrimerACGCTTCACACGTTCGGATGAG; Reverse Primer TGACAGGTGGTCACTCCTCATG human S1P: Forward Primer CTACTATGGAGGAATGCCGACAG; Reverse Primer CTCCGTTCTGTGGCAAATAGGG human S2P: Forward Primer ACGGCGGAAAGCAAGGATGCTT; Reverse Primer GTGCCAAAGTCTGCATCAGCGT human GAPDH: Forward Primer GTCTCCTCTGACTTCAACAGCG; Reverse Primer ACCACCCTGTTGCTGTAGCCAA.

### 2.3 Western blot

Total protein was extracted by suspending the cell pellet in a cell lysis buffer containing 50 mM Tris‐HCl (pH 7.4), 150 mM NaCl, 1% Triton X‐100, 0.1% SDS, and 1 mM EDTA supplemented with protease inhibitor cocktail from Roche (Sigma, St. Louis, MO) (Brown et al., 2011). The BCA kit (Abcam, ab102536) was used to determine the protein concentration. Protein (100 μg) from both control and treated samples was subjected to 4%–20% SDS‐PAGE. The resolved proteins in the gel were transferred to a PVDF membrane electrophoretically. The membrane was separately incubated first with Anti‐SREBP2 antibody (ab30682), Anti‐SCAP antibody (ab190103), Anti‐SP1 antibody (ab227383), Anti‐S1P antibody (ab140592), Anti‐S2P antibody (ab140594), Anti‐GAPDH antibody (ab181602) from Abcam (1:1,000) and then with goat anti‐rabbit secondary antibody (1:10,000) conjugated with horseradish peroxidase followed by ECL detection from Bio‐Rad (Hercules, CA).

### 2.4 Immunofluorescence

The cells were 4% paraformaldehyde fixed (15min) at room temperature and then incubated with 0.1% Triton X‐100 in PBS for 15min to permeabilize the cells and block nonspecific protein‐protein interaction with 5% BSA for 30min. The cells were then incubated with the antibody SREBP2 (1:100) overnight at 4℃. The secondary antibody (green) Alexa Fluor 488 goat anti‐rabbit IgG (H + L) (1:200) was incubated 2h at RT; then, ER‐tracker Red Beyotime (C1041) was used to label ER membranes (red) at a 1:200 dilution for 30min. DAPI Beyotime (C1005) (1:1,000) was used to stain the cell nuclei (blue) for 5 min.

### 2.5 Statistics

**FIGURE 1:**
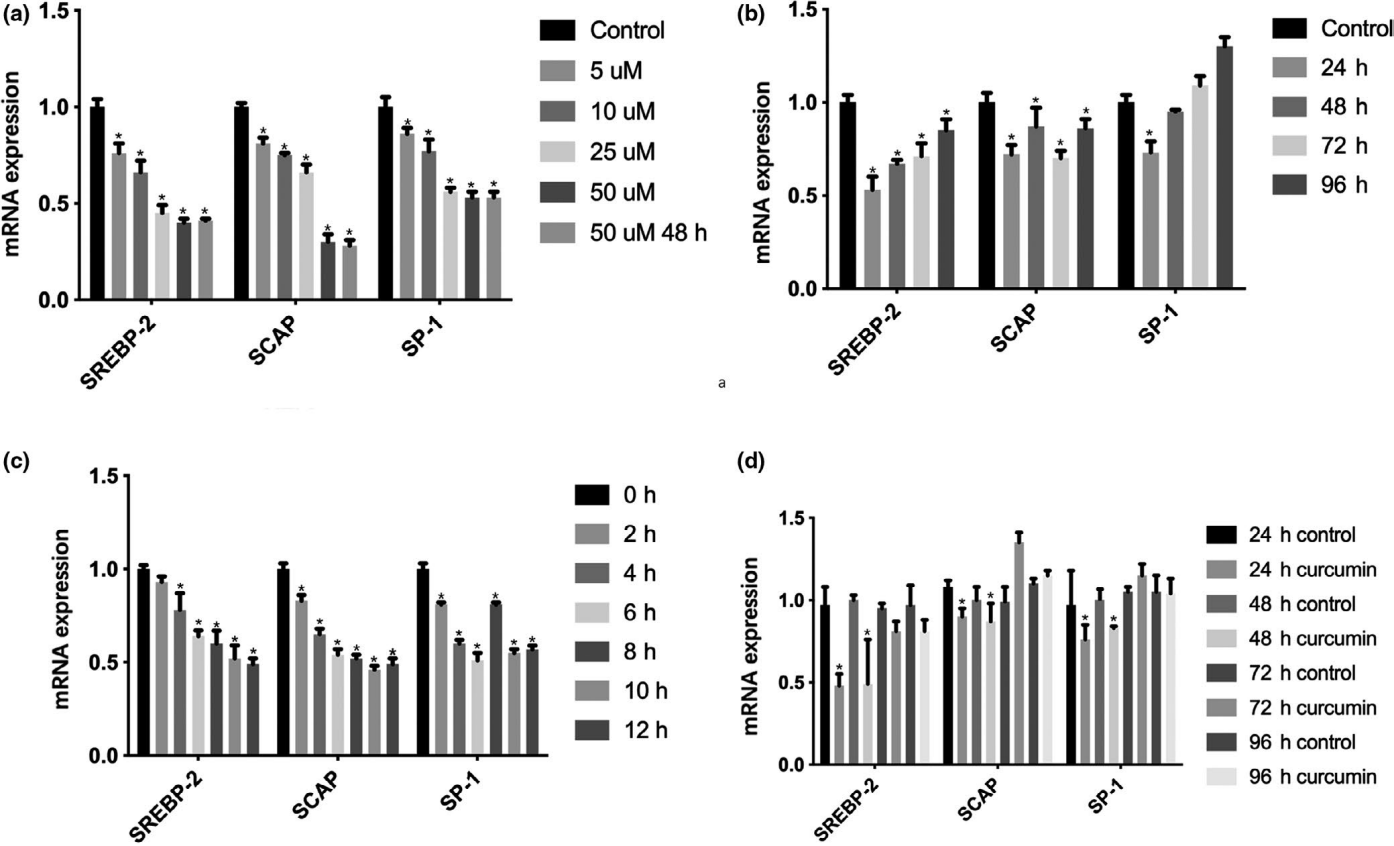
Dose–response and time effects of curcumin on the mRNA expression of SREBP‐2, SP‐1, and SCAP. (a) mRNA expression at 24 and 48 hr cultivation in different concentrations of curcumin *, *p* < .05, versus the untreated control. (b) mRNA expression under 25 μM curcumin treatment from 0 ~ 96 hr *, *p* < .05, versus the untreated control. (c) mRNA expression under 25 μM curcumin treatment from 0 ~ 12h.*, *p* < .05, versus the untreated control. (d) The comparison of mRNA expression in control group and curcumin group at each time point. *, *p* < .05, versus the untreated control in each point‐in‐time. Sketch Map. Normally, INSIG binds to the sterol‐sensing domain (SSD) of SCAP (blue transmembrane region in SCAP). As the sterols concentration decreases, the connection between SCAP and INSIG disappears, and the complex of SREBP/SCAP is transported from the endoplasmic reticulum to the Golgi apparatus. S1P and S2P were sequentially involved in cleaving SREBP‐2 and releasing the mSREBP‐2 into the cell nucleus

**FIGURE 2:**
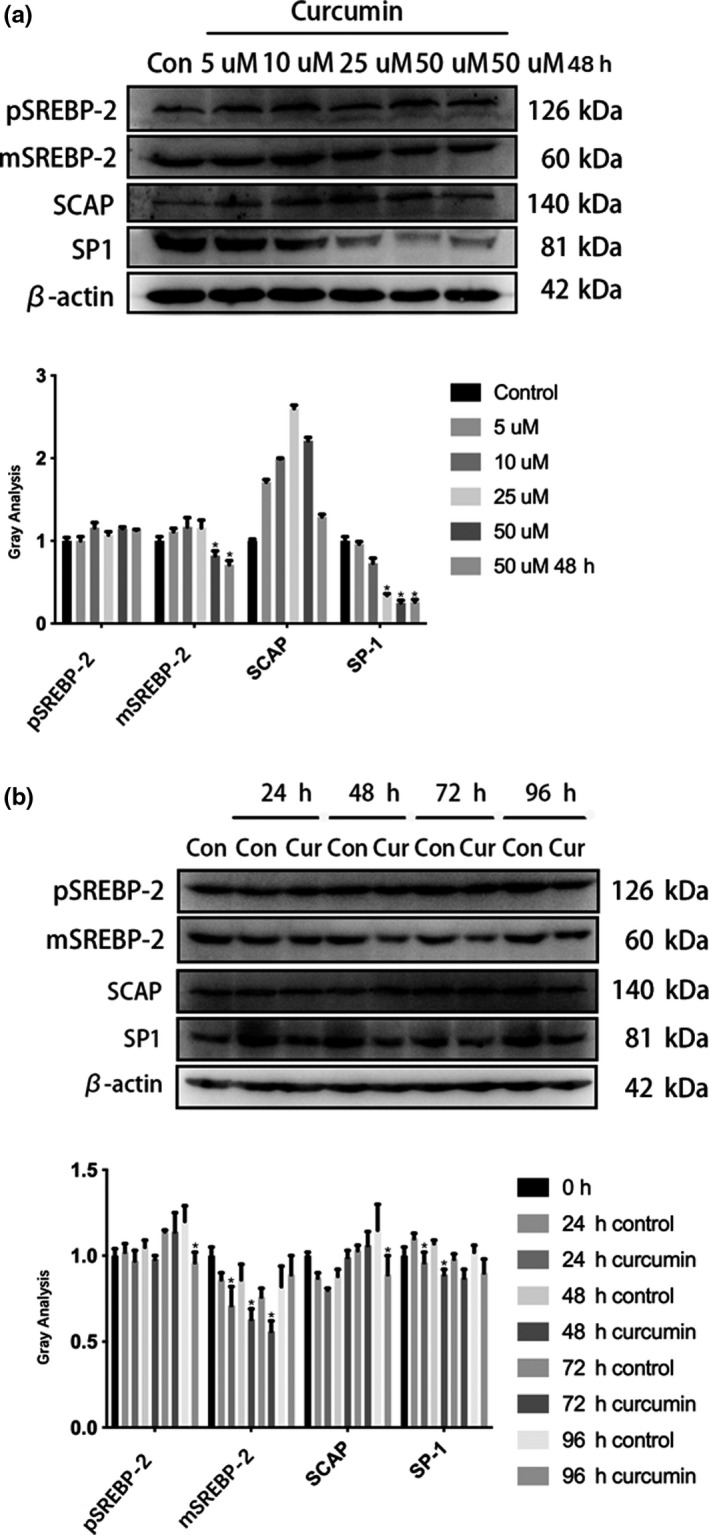
In Western blotting analysis, the precursor and mature SREBP‐2, SP‐1, and SCAP in cells were treated with curcumin at different concentrations. β‐actin was used as an invariant control for equal loading. Representative was shown from three independent experiments. a, The detection of protein expression and the gray value analysis under different concentrations of curcumin treatment at 24 hr and under 50 μM curcumin treatment at 48 hr *, *p* < .05, versus the untreated control; #, *p* > .05, versus the untreated control. b, The expression of protein and the gray value analysis under 25 μM curcumin treatment from 0 to 96 hr cultivation. *, *p* < .05, versus the untreated control; #, *p* > .05, versus the untreated control

**FIGURE 3:**
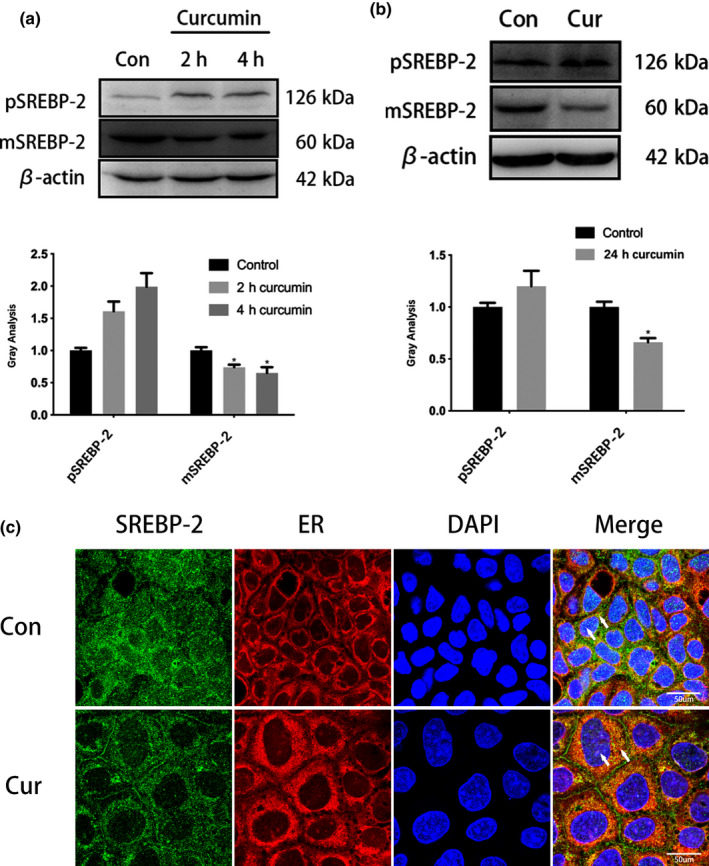
The intracellular distribution changes of precursor and mature SREBP‐2 incubated for different time (0‐24 hr) under curcumin intervention. (a) The expression changes of precursor and mature SREBP‐2 in the cytoplasm and nucleus of precursor and mature SREBP‐2 for different time(0‐4 hr) under 25 μM curcumin intervention was detected by Western blot, and the gray intensity was analyzed. *, *p* < .05, versus the untreated control; #, *p* > .05, versus the untreated control. (b) Western blot was used to detect the protein expression in the cytoplasm and nucleus of precursor and mature SREBP‐2 after 24 hr cultivation under 25 μM curcumin intervention. *, *p* < .05, versus the untreated control; #, *p* > .05, versus the untreated control. (c) The images of co‐localization of SREBP‐2 in endoplasmic reticulum after 24 hr cultivation under 25 μM curcumin intervention. Green fluorescence indicates the SREBP‐2, red fluorescence indicates the endoplasmic reticulum, and blue fluorescence indicates the cell nucleus. The white arrow indicates the distribution of SREBP‐2 in Caco‐2 cells

**FIGURE 4:**
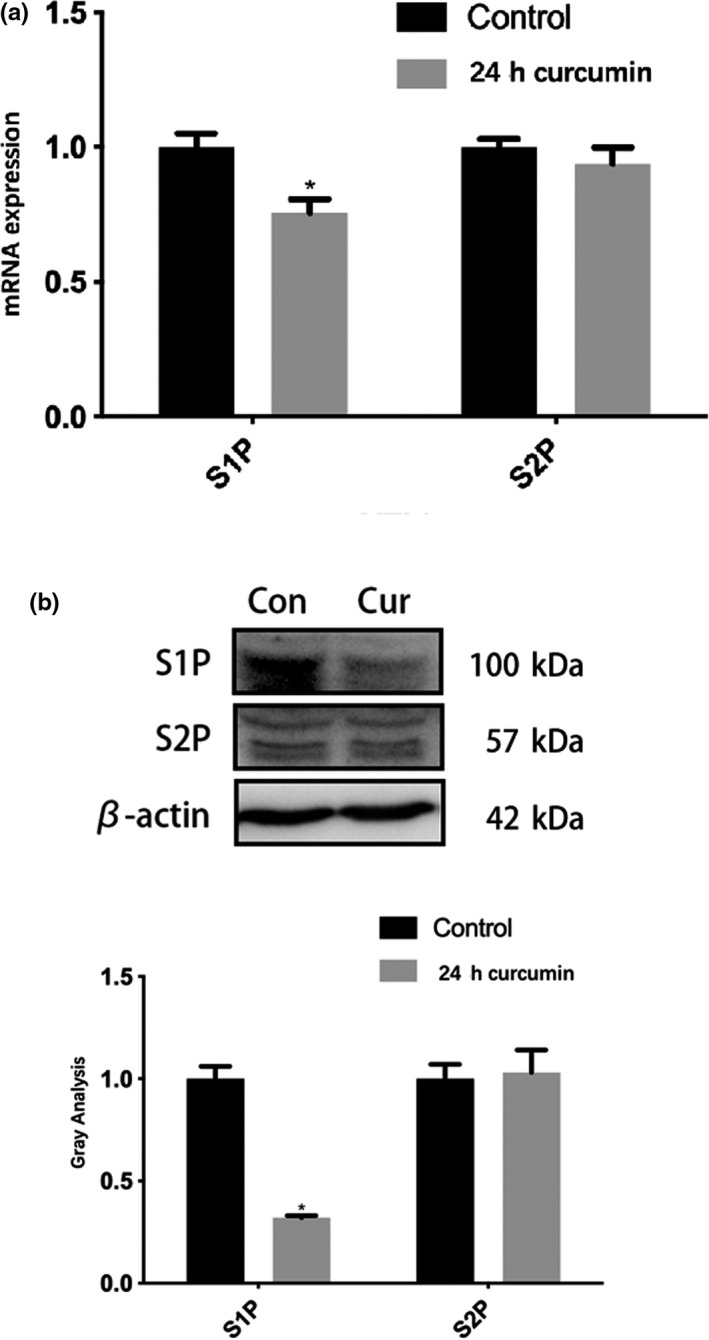
The expression of S1P/S2P mRNA and protein under 25 μM curcumin treatment. (a) The change of mRNA expression. *, *p* < .05, versus the untreated control (first column). (b) The Western blot and the gray value analysis results. β‐actin was used as an invariant control for equal loading. *, *p* < .05, versus the untreated control (first column)

The results of multiple concentrations and multiple time points were statistically analyzed using one‐way analysis of variance (Figure 1a‐d, Figure 2a, Figure 2b, Figure 3a). A *p* value of .05 or less was considered statistically significant. Other experimental groups were statistically analyzed by Student's *t* test (Figure 3b, Figure 4a, Figure 4b). A *p* value of .05 or less was considered statistically significant.

## 3 RESULTS

### 3.1 Curcumin inhibits the expression of SREBP‐2, SCAP, and SP‐1

Many studies report inhibited expression of SREBP‐2 mRNA upon curcumin treatment (Brown et al., 2017; Ding et al., 2016; Kang & Chen, 2009; Kang & Chen, 2009; Kumar et al., 2011; Li et al., 2015; Liu et al., 2017). Our study showed consistent results, with mRNA levels of SREBP‐2, SCAP, and SP‐1 mRNA decreasing as the curcumin concentration increased. With 50 μM curcumin, the mRNA levels of SREBP‐2, SCAP, and SP‐1 mRNA were not significantly different at 48 hr compared to 24 hr There was also no significant change in levels of SREBP‐2 and SP‐1 mRNA when the curcumin concentration was 25 μM or 50 μM (Figure 1a). Thus, we assayed the time course of curcumin at a 25 μM concentration. As shown in Figure 1b, mRNA levels for SREBP‐2, SCAP, and SP‐1 were initially inhibited, yet this inhibition lessened with prolonged treatment. Levels of SP‐1 were seen to completely recover by 48 hr, while SREBP‐2 and SCAP mRNA levels were slightly lower in treated cells even at 96h. We also found that the expression of SREBP‐2, SCAP, and SP‐1 mRNA were downregulated by curcumin treatment at 12 hr, with SP‐1 levels decreasing faster than SREBP‐2 or SCAP (Figure 1c). This strongly supports the study by Kang et al identifying that SP‐1 acts as a transcription factor binding to the GC‐box on the SREBP‐2 promoter to regulate SREBP‐2 gene expression (Kang & Chen, 2009). To diminish the effects of cell proliferation, we set up control groups at each time point. We found that the expression of SREBP‐2 and SCAP was lower with curcumin than in the control group. SP‐1 expression was inhibited at 24 hr under curcumin treatment, and the effect was eliminated after 48 hr, consistent with the time and effect of curcumin in Kang & Chen (2009) (Figure 1d).

### 3.2 Curcumin did not simultaneously downregulate the expression of SREBP‐2 and SCAP proteins

After treatment with curcumin in Caco‐2 cells, although SREBP‐2, SCAP, and SP‐1 mRNA expression were downregulated in both dose‐effect and time‐effect tests, there was no simultaneous downregulation of protein expression. As shown in Figure 2a, at 24h treatment with an increased concentration of curcumin, the levels of pSREBP‐2 (130 kDa) and SCAP showed no significant difference compared to the control group, yet mSREBP‐2 protein (55‐70 KDa) was decreased. SP‐1 expression gradually decreased with increasing curcumin concentration, but no change in SP‐1 expression was seen after 48h treatment. The time course results showed that the protein levels of pSREBP‐2 and SCAP did not decease although their mRNAs were downregulated following curcumin treatment. The pSREBP‐2 and SCAP protein levels showed no significant change compared to the control group at 72 hr treatment. However, at 96h treatment, the levels of pSREBP‐2 and SCAP protein were decreased. The level of mSREBP‐2 mature protein was also decreased upon curcumin treatment compared to the control group (Figure 2b). The observation that curcumin treatment did not decrease the level of pSREBP‐2 while mSREBP‐2 protein levels were downregulated suggest that curcumin may inhibit proteolysis of the pSREBP‐2.

### 3.3 Curcumin inhibits the proteolysis of the SREBP‐2 precursor and decreases the level of SREBP‐2 mature protein

To test this hypothesis, we examined the distribution of SREBP‐2 in the cytoplasm and nucleus early in curcumin treatment. As shown in Figure 3a, mature SREBP (55‐70kDa) decreased in curcumin treatment, but the pSREBP‐2 (130 kDa) did not decrease with prolonged treatment. After 24 hr curcumin treatment, Western blotting showed that the amount of pSREBP‐2 in the cytoplasm was not significantly different from that of the control group, while the amount of mSREBP‐2 protein in the nucleus was less than in the control group (Figure 3b). Tracking co‐localization of SREBP‐2 protein with the endoplasmic reticulum showed that the amount of mSREBP‐2 in the nucleus was reduced and more pSPEBP‐2 stayed in the cytosol and endoplasmic reticulum after curcumin intervention (Figure 3c).

### 3.4 Curcumin may downregulate the expression of S1P

To explore the mechanism of curcumin inhibiting proteolysis of the pSREBP‐2, we examined the expression of S1P and S2P relative to SREBP‐2 proteolysis after curcumin intervention. We found that after 24 hr treatment, 25 μM curcumin could downregulate the expression of S1P but not S2P in CaCo‐2 cells (Figure 4). Therefore, we speculate that curcumin may affect the proteolysis process of SREBP‐2 by inhibiting the expression of S1P.

## 4 DISCUSSION

Curcumin is a chemical component extracted from the rhizome of Zingiberaceae and Araceae. Studies have described various effects of curcumin including reducing blood fat, choleretic actions, and antitumor, anti‐inflammatory, and antioxidation effects (Moghaddam et al., 2019; Nelson et al., 2017; Panahi et al., 2018; Sato et al., 2013). Several studies have indicated that the effect of curcumin on SREBPs is related to the mechanism of curcumin regulating blood lipid metabolism (Brown et al., 2017; Ding et al., 2016; Kang & Chen, 2009; Kang & Chen, 2009; Kumar et al., 2011; Li et al., 2015; Liu et al., 2017). Kumar et al (Brown et al., 2011) found that curcumin can regulate the expression of NPC1L1 through SREBP‐2 to inhibit cholesterol absorption in Caco‐2 cells. We used a mouse model of cholesterol gallstone formation induced by a high‐fat diet to show these results. Our preliminary study further confirmed that curcumin can reduce cholesterol gallstone formation and inhibit NPC1L1 (Ding et al., 2015). As we investigated how curcumin affects NPC1L1 expression through SREBP‐2, we found different results from previous studies. Although curcumin lowered SREBP‐2 mRNA levels, as in other studies, we did not see simultaneously downregulated SREBP‐2 protein levels. In the Caco‐2 cell line, SREBP‐2 mRNA expression decreased with higher doses (Figure 1a) and times (Figure 1b) of curcumin treatment. However, we did not find pSREBP‐2 precursor levels to be inhibited (Figure 2). Western blotting showed that while pSREBP‐2 (almost 130kDa) was not significantly downregulated, the amount of mSREBP‐2 protein (55‐70kDa) decreased (Figure 2). The SREBP‐2 mRNA levels were inconsistent with pSREBP‐2 levels, which was particularly evident through the curcumin time course. Regardless of whether curcumin was administered long‐term (0 ~ 72h) or short‐term (within 24h), the pSREBP‐2 did not decrease following with the mRNA inhibition. Kang and Chen (2009) found that the curcumin‐mediated reduction of SREBP‐2 promoter activity was dependent on the inhibition of SP‐1. Consistent with Kang's study, we also found downregulated transcriptional and translational levels of SP‐1 at 24 hr treatment, but this downregulation was relieved after 48 hr treatment (Figure 1b, Figure 2b). This suggests that curcumin's effect on SREBP‐2 may not involve SP‐1 or that there may be another pathway. Considering the amount that mSREBP‐2 protein decreased after curcumin treatment and that SREBP‐2 is activated as a transcription factor through the proteolytic process (Feng et al., 1999; Field et al., 2018), we hypothesized that the effect of curcumin on SREBP‐2 may depend on its proteolytic process. Endoplasmic reticulum co‐localization showed that curcumin modulated SREBP‐2 distribution within the cell, decreased mSREBP‐2 in the nucleus, and inhibited the proteolysis process (Figure 3c). In Brown and Goldstein's research (Feng et al., 1999; Field et al., 2018), they suggest that the SREBP‐2/SCAP complex is the real substrate of S1P and key for the proteolytic process of SREBP‐2. Therefore, we also detected SCAP in our study. SCAP and SREBP‐2 showed consistent changes in transcription and translation. These results suggest that SCAP may not be a target of curcumin. As S1P protein levels decreased after curcumin treatment, curcumin may inhibit the expression of SREBP‐2 by inhibiting the expression of S1P.

Several other studies have demonstrated inconsistency between SREBP‐2 transcription and translation levels. Field et al (Wang et al., 2001) showed in rat liver cells that increased cholesterol in Caco‐2 cells inhibited proteolysis of SREBP‐2. The mSREBP‐2 was decreased but pSREBP‐2 levels were not significantly changed, and this process was accompanied by inhibition of SREBP‐2 gene expression. Liu et al (Kumar et al., 2017) reported that the change of SREBP‐2 protein was not statistically significant after curcumin treatment compared with controls. Sato et al (Zingg et al., 1996) found that the SREBP‐2 gene includes a sterol regulatory element (SRE) identical to the one on the promoter sequence of the human LDL receptor; thus, SREBP‐2 may regulate its own expression through modulating sterol levels. When the proteolytic process of SREBP‐2 was inhibited, mSREBP‐2 transcription factor levels decreased, which affected the expression of cholesterol metabolism‐related proteins and also the expression of SREBP‐2. These results may explain why the expression of SREBP‐2 mRNA does not follow the protein level.

Some studies have reported downregulated SREBP‐2 upon curcumin treatment (Kang & Chen, 2009; Li et al., 2016; Liu et al., 2017). However, these studies do not show the molecular weight of the protein markers in their figures, making it hard to determine the molecular weight of the SREBP‐2 detected in their Western blots. Thus, the detected protein may be mSREBP‐2 rather than pSREBP‐2. Liu et al (Kumar et al., 2017) studied the function of curcumin using antibodies against SREBP‐2 purchased from Santa Cruz which have only been used to detect pSREBP‐2. Our results indicate that curcumin first affects the SREBP‐2 proteolysis process rather than inhibiting its protein expression. Protein expression inhibition may be a long‐term effect. The inhibitory effect of curcumin on the transcription of SREBP‐2 mRNA persisted long‐term, with decreased mRNA of the pSREBP‐2 detected after 96 hr treatment (Figure 2b). When SREBP‐2 proteolysis is inhibited, pSREBP‐2 may be degraded by other pathways, and its gene expression may remain continuously inhibited, eventually leading to downregulation of the pSREBP‐2 protein expression. This mechanism still requires further experimentation to fully understand.

The exact mechanism for how curcumin reduces the hydrolysis of SREBP‐2 and deceases mSREBP‐2 protein levels through inhibiting S1P expression remains unknown. However, we conclude here that the mechanism of curcumin treatment on SREBP‐2 is not through short‐term inhibition of protein expression, although curcumin can indeed inhibit the transcription of SREBP‐2 mRNA long‐term. Thus, it appears that curcumin plays an important role via inhibiting the activity of SREBP‐2 rather than directly affecting its expression of gene and protein. In this study, we propose that curcumin may decrease the amount of mSREBP‐2 by directly inhibiting the expression of S1P mRNA and protein. To our knowledge, this result is the first to be reported. The conclusion of this study provides a new idea and target for exploring the mechanism of curcumin inhibiting cholesterol absorption. At the same time, this study also promotes the clinical application of curcumin in the prevention of gallbladder cholesterol gallstones.

## 5 CONCLUSION

Curcumin can inhibit the SREBP‐2 proteolytic process to reduce mSREBP‐2 which functions as a transcription factor, affecting the regulation of cholesterol metabolism‐related genes. This process may be achieved by regulating the expression of S1P. Curcumin does not directly inhibit the expression of mSREBP‐2 protein, and it has no such inhibitory effect for at least a short period of time, although curcumin does reduce the amount of mSREBP‐2 protein.

## CONFLICT OF INTEREST

None.
